# Research on Geometric Calibration of Spaceborne Linear Array Whiskbroom Camera

**DOI:** 10.3390/s18010247

**Published:** 2018-01-16

**Authors:** Qinghong Sheng, Qi Wang, Hui Xiao, Qing Wang

**Affiliations:** 1 College of Astronautics, Nanjing University of Aeronautics and Astronautics, Nanjing 210016, China; qiwang@nuaa.edu.cn (Q.W.); xinyue@nuaa.edu.cn (Q.W.); 2 University School of Environmental Science, Nanjing Xiaozhuang University, Nanjing 211171, China; xiaohui@njxzc.edu.cn

**Keywords:** thermal-infrared camera, spaceborne linear array whiskbroom, imaging geometric model, geometric calibration

## Abstract

The geometric calibration of a spaceborne thermal-infrared camera with a high spatial resolution and wide coverage can set benchmarks for providing an accurate geographical coordinate for the retrieval of land surface temperature. The practice of using linear array whiskbroom Charge-Coupled Device (CCD) arrays to image the Earth can help get thermal-infrared images of a large breadth with high spatial resolutions. Focusing on the whiskbroom characteristics of equal time intervals and unequal angles, the present study proposes a spaceborne linear-array-scanning imaging geometric model, whilst calibrating temporal system parameters and whiskbroom angle parameters. With the help of the YG-14—China’s first satellite equipped with thermal-infrared cameras of high spatial resolution—China’s Anyang Imaging and Taiyuan Imaging are used to conduct an experiment of geometric calibration and a verification test, respectively. Results have shown that the plane positioning accuracy without ground control points (GCPs) is better than 30 pixels and the plane positioning accuracy with GCPs is better than 1 pixel.

## 1. Introduction

A spaceborne thermal-infrared camera is characterized by its advantages in Earth observation with its clearness in target details, its strong capability in target identification [1,2,3], and its capability of being used in all-weather conditions, especially in adverse weather conditions [4,5]. Therefore, high spatial resolution and wide ground coverage are the developing trends of spaceborne thermal-infrared imaging [6]. The thermal-infrared satellite’s remote sensing with high spatial resolution can measure the land surface temperature on a comparatively small spatial scale and when compared with the traditional thermal-infrared satellite image with low spatial resolution, the former has the features of being more informative; more evident in target temperature and geometric structure, and so forth [7,8,9].

In the technology of spaceborne thermal-infrared imaging with high resolutions, the multi-spectral scanner (MSS) and enhanced thematic mapper (ETM) of the early Landsat series adopted the method of object space scanning by point whiskbroom to obtain thermal-infrared images. Admittedly, by this, an image with higher resolution could be obtained, but the size of the object lens was comparatively larger, and the optical camera had a heavy and complex mechanical structure. Nowadays, the CCD pushbroom imaging technology tends to be the more popular choice in the Earth observation field to be used by satellites with high resolution. The spaceborne linear array CCD pushbroom imaging has been widely used in thermal-infrared sensors (TIRS), advanced spaceborne thermal emission and reflection radiometers (ASTER) and in the Micro-Trak II (MTI) sensors. The image quality of the spaceborne linear array CCD pushbroom camera is high, and the structure of the optical machine is simple. However, thermal-infrared CCD includes a photosensitive detector that has a small number of units, and the field of view of a single image is small, only getting a small range of target information. For example, the number of CCD in the MTI thermal-infrared band is 208, with a spatial resolution of 20 m, but a swath width of only 12 km. The YG-14 is China’s first satellite equipped with thermal-infrared cameras with high spatial resolution and wide coverage. While the satellite is flying along the track, the scanning mirror of the camera can conduct an oscillatory scanning while it revolves around the direction along the track simultaneously. Moreover, the field of view increases with an increase of the scanning angle, with its maximal view reaching 4°, with a width of 70 km, and a spatial resolution of 10 m. The on-orbit geometric calibration is the key to guaranteeing the geometric quality of the YG-14 thermal-infrared satellite’s image and the elimination of the errors of an on-orbit imaging system.

The external calibration and the internal calibration of the linear array whiskbroom camera are the same with the pushbroom camera. The pushbroom camera, in terms of geometric calibration, makes use of offset matrix [10] to compensate for the direction deviation between the actual imaging ray and the ideal imaging ray. Additionally, the use of a few control points eliminates the systematic errors such as the attitude and orbit measurement error, the measuring equipment error, the camera installation angle error, and so forth. Using the detector look angle as an interior orientation element to establish polynomial modeling [11] uniformly reflects the effect of the principal point deviations, the error of focal length, lens distortion, and so forth, on the imaging geometry so as to calibrate the error of each detector look angle, and as a result, eliminate the errors of the camera’s interior orientation elements. Therefore, the imaging geometric model of the linear array whiskbroom camera may use an offset matrix to eliminate the errors of exterior orientation elements and use the detector look angle to eliminate the errors of the interior orientation elements.

There are also some differences between the linear array whiskbroom camera and the pushbroom camera. In wide coverage imaging, new errors will be introduced in the spaceborne linear array whiskbroom camera. Firstly, in the auxiliary data of the YG-14 thermal-infrared linear array whiskbroom, each scanning cycle only provides the imaging time of a certain specific scanning line and the line number of specific scanning lines in different scanning cycles is different. Therefore, it is impossible to accurately obtain the three time quanta related to camera imaging: the time start, the line integration time and the imaging cycle during each scanning cycle. The geometric model that describes the mapping relationship between a two-dimensional image plane and a three-dimensional object space mapping is related with time. If the time system is not unified, there will be a decline in the internal stability of the imaging model along the track [12]. Secondly, because of influences such as thermal deformation, weightlessness and so on, the vertical scanning of the scanning mirror by mechanical control cannot strictly swing at the same angle [13,14]. The starting angle is different from the calibration value set in the lab, and the uneven whiskbroom angle leads to an accuracy decline in the imaging model across the track.

The linear array whiskbroom system, mainly concentrated within the airborne field such as in hyperspectral imaging, uses an equiangular control model at the boundary of the whiskbroom [15,16,17]. This is different from uniformly setting the whiskbroom angle in the airborne system as the whiskbroom angular velocity is nonlinear, which leads to a non-uniformity of line integration time.

To make use of the geometric model by spaceborne linear array CCD pushbroom imaging to conduct external calibration and internal calibration whilst noting the special error characteristics of the whiskbroom camera, the present paper is aimed at on-orbit geometric calibration in regard to imaging time start, line integration time, imaging the start angle and line whiskbroom angle. In the second part of this paper, for the sake of eliminating the errors along and across the track, the imaging time start and the line integration time are collectively referred to as the time system parameters, and the imaging starting angle and the line whiskbroom angular velocity are referred to as the whiskbroom angle parameters. In the third part, China’s Anyang data is chosen for geometric calibration, and the calibration parameter is verified by making use of China’s Taiyuan data.

## 2. Methods

**Figure 1:**
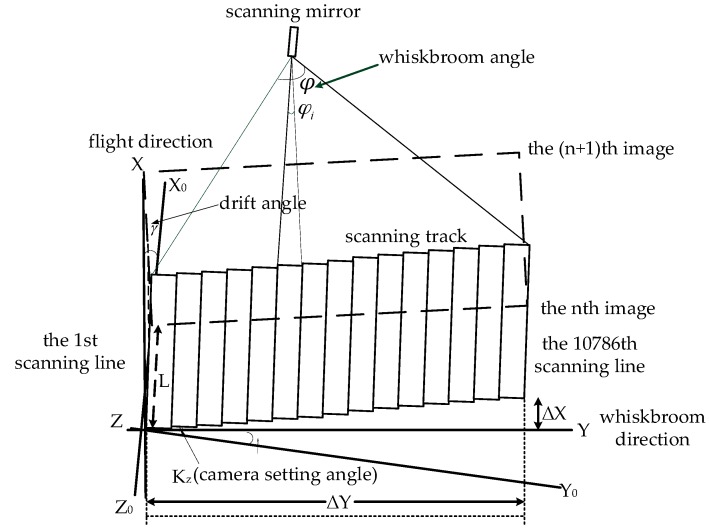
The scanning image of the thermal-infrared linear array whiskbroom camera.

**Figure 2:**
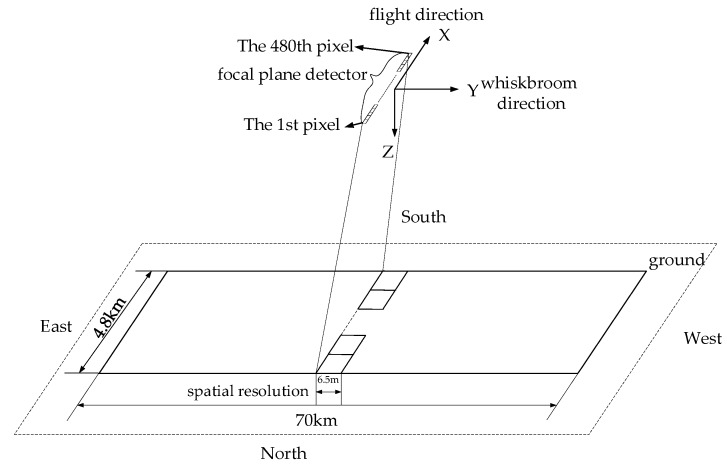
The diagram of the ground projection position of the imaging detector pixel.

Figure 1 shows the scanning ground image made by the thermal-infrared linear array whiskbroom camera of the YG-14. While the satellite is flying, the scanning mirror is swinging to get a thermal-infrared image. The displacement along the track ΔX is influenced by the satellite’s flying velocity, the component of the camera-setting angle along the track and the component of the drift angle along the track. The displacement across the track ΔY is obtained by the speed of the whiskbroom angle, the component of camera setting angle across the track and the component of the drift angle across the track [18]. Figure 2 shows the ground projection position of the imaging detector pixel. The size of the instant imaging of the spaceborne linear array CCD is about 6.5 m × 4.8 km—named as one subframe—and the scope of the whiskbroom angle is ±2.0875°. A total of 10,786 thin and long strips, obtained in one scanning cycle, are spliced together to form an image with a scope of 70 km × 4.8 km. It is obvious that the major difference between the linear array whiskbroom imaging and the linear array pushbroom imaging lies in the CCD arrangement that is changed from a direction across the track to one that is along the track. Furthermore, the scanning mirror revolves around the *X* axis of the sensor coordinate system.

### 2.1. Equal Time Intervals and Unequal Angular Whiskbroom Characteristics

#### 2.1.1. The Whiskbroom Characteristics of Equal Time Intervals

**Figure 3:**
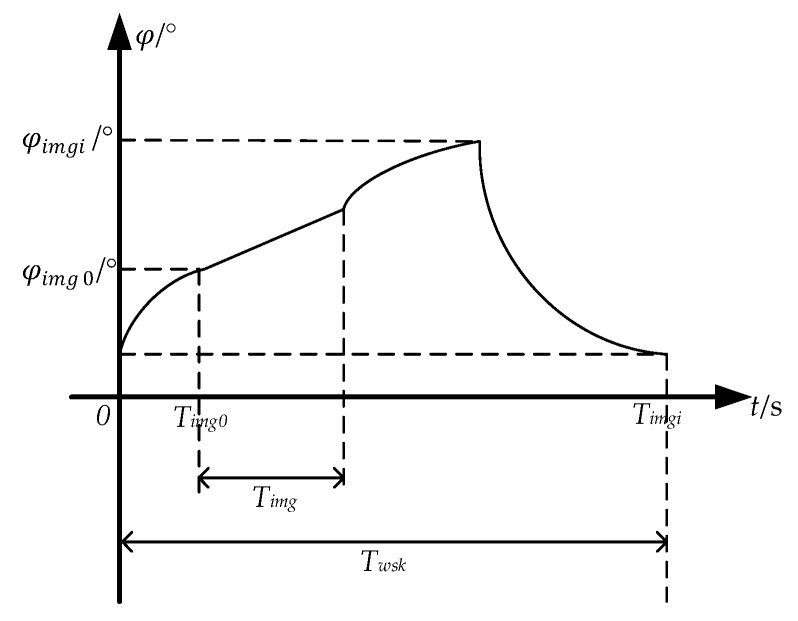
The whiskbroom angle curve in a scanning cycle.

**Figure 4:**
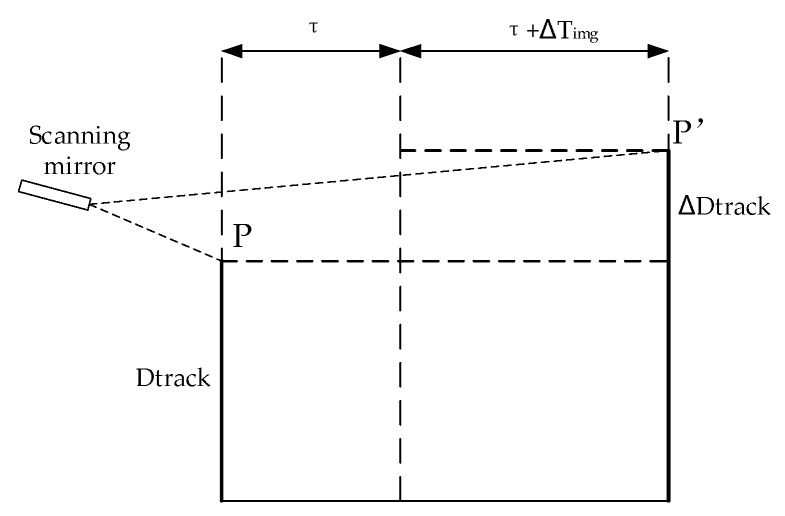
The diagram of deviation along the track caused by the imaging time error.

As shown in Figure 3, in a whiskbroom cycle $T_{wsk}$, the scanning mirror starts to change the speed of the whiskbroom from its mechanical limit until it reaches the designated speed and then begins to form the image, with the imaging time start set as $T_{img0}$. Now the scanning mirror is approximately in uniform motion before the end of the imaging cycle $T_{img}$, and then the camera lens is closed and the scanning mirror speeds back up to its starting mechanical limit to enter the next whiskbroom cycle. Figure 4 shows the deviation along the track caused by the imaging time error. In two adjacent whiskbroom cycles, the ground coverage of the same detector on the same whiskbroom line along the track is set as $D_{track}$, and due to the change of the actual state of the mechanical process and the control system after the satellite enters the orbit, a deviation between the imaging time start $T_{img0}$ and the calibration value (set in the lab) occurs, leading to the imaging deviation along $\Delta D_{track}$:

(1) $$\Delta D_{track}\;=\;\frac{D_{track}\cdot \Delta T_{img}}{T_{img}}$$

In Equation (1), $\Delta T_{img}$ is the deviation between the actual imaging cycle and the calibration cycle set in the lab. Supposing the whiskbroom takes place at equal intervals in imaging, the equation will be:

(2) $$T_{img}\;=\;T_{img0}+\tau \cdot L_{wsk},$$

In Equation (2), τ is set as line integration time, and $L_{wsk}$ is set as the whiskbroom line number in an imaging cycle $T_{img}$.

The imaging cycle is related to the imaging time start of each image, and therefore the calibration should be decided from the first image. Since the first whiskbroom line of the first image is $T_{img0}$ ≈ 10^−9^ s, it can be ignored. Then, Equation (2) is put into Equation (1):

(3) $$\Delta D_{track}\;=\;\frac{D_{track}\cdot \Delta T_{img}}{\tau \cdot L_{wsk}}$$

$D_{track}$ is set as 480 pixels and $L_{wsk}$ as line number 10,786, and therefore for any one imaging cycle, when its time deviation $\Delta T_{img}$ is one line integration time, the deviation occurring along the track will be only 0.0445 pixels. When the satellite is in an on-orbit operation, with the imaging cycle deviation $\Delta T_{img}$ being less than 2 line integration time, its effect on the positioning accuracy is less than 1 pixel. Therefore, the whiskbroom imaging of the YG-14 is characterized by equal time intervals.

#### 2.1.2. The Whiskbroom Characteristics of Unequal Angle

Figure 3 shows the whiskbroom angle curve in a scanning cycle. Since the imaging start angle $\varphi_{img0}$ and the angular velocity $\omega_{yj}$ of each whiskbroom line determine the size of the whiskbroom angle at the end image of each line, the whiskbroom angle $\varphi_{imgi}$ at the imaging end of the line *i* is:

(4) $$\varphi_{imgi}\;=\;\varphi_{img0}+\sum_{j\;=\;0}^{i}\omega_{yj}\cdot \tau \;\left(i\;=\;1,2,\ldots ,\;L_{wsk}\right),$$

Equations (2) and (4) show that the imaging geometric model of the linear array whiskbroom camera needs to calibrate the imaging time start $T_{img0}$, the line integration time τ, the imaging start angle $\varphi_{img0}$, and the line whiskbroom angular velocity $\omega_{yj}$. Among them, $T_{img0}$ and τ are both time system parameters, leading to an image distortion along the track. In the imaging geometric model, the satellite’s position [*X_S_ Y_S_ Z_S_*]^T^ in the World Geodetic System 1984 (WGS84) coordinate system; the rotation matrix $R_{body}^{J2000}$ measured by the orientation system between the body coordinate system and the J2000 coordinates; and the whiskbroom angle $\varphi_{imgi}$ at the imaging end of each line, are all related to the time system. If all the related time parameters do not agree, the measured track data and the attitude measurement data for a specific scan line will not be able to correspond to the line whiskbroom angle. Only when the time systems are made uniform, can the stability of the imaging geometric model be ensured. $\varphi_{img0}$ and $\omega_{yj}$ are the whiskbroom angle parameters, leading to image distortion across the track. Therefore, the calibration of angle parameters can improve the local accuracy of the model, avoiding image distortion such as serrated and other aberrations.

### 2.2. The Time System Parameter Calibration of the Spaceborne Linear Array Whiskbroom

#### 2.2.1. The Calibration of the Imaging Time Start

The present paper makes use of the imaging geometric model that has been externally calibrated to resample the control images, ensuring that not only the control image and the original thermal-infrared image to be calibrated are in agreement in spatial resolution but also that the homonymy point coordinates are exactly the same. However, homonymy points do not necessarily correspond to homonymy detectors because an imaging model that has been externally calibrated would not have eliminated the error made by the whiskbroom motion and the error of the interior orientation elements. Because the imaging time *T_L_* of the first whiskbroom line is less than 10^−9^ s, it can be approximated as *T_L_* = *T_img_*_0_. Therefore, the calibration of the imaging time start *T_img_*_0_ can be converted to the calibration of the imaging time of the first whiskbroom line. Suppose that *N_pxl_* is the total number of pixels for each whiskbroom line, along the track of the original thermal-infrared image. When the offset in the pixel of the first whiskbroom line and the same pixel of the control image are set as Δ*x_j_* (*j* = 0, 1, …, *N_pxl_*) and the velocity of satellite flight is set as *V_x_*, the start time of imaging *T_img_*_0_ will be:

(5) $$T_{img0}\;=\;T_{L}\;=\;\frac{\sum_{j\;=\;0}^{N_{pxl}}\Delta x_{j}}{N_{pxl}\cdot V_{x}},$$

#### 2.2.2. The Calibration of the Line Integration Time

The displacement along the track ΔX of the whole scene in an imaging cycle is determined by the component of camera detector angle around the *Z* axis $K_{zx}$, the rotation of the earth drift angle $\gamma_{x}$ and the satellite flight velocity $V_{x}$, and thereby such an equation can be established as:

(6) $$\Delta X\;=\;A_{x}\cdot \frac{V_{x}}{\cos\gamma_{x}}T_{img}\cos K_{zx},$$

In Equation (6), $A_{x}$ is the mapping scale of the image along the track. In two consecutive images, supposing that the same detector coordinates are (*x*_1_, *y*_1_) and (*x*_2_, *y*_2_), then the image motion along the track will be $\Delta X\;=\;\left|x_{2}-x_{1}\right|$. $\Delta T_{img}$ and $\Delta K_{zx}$, the correction values of $T_{img}$ and $K_{zx}$, are set as an unknown number to establish error equations and the normal equation in order to get the least-square solutions, thus calibrating the imaging cycle $T_{img}$ and accordingly getting the line integration time $\tau \;=\;\frac{T_{img}}{L_{wsk}}$.

### 2.3. The Angle System Parameter Calibration of the Spaceborne Linear Array Whiskbroom

**Figure 5:**
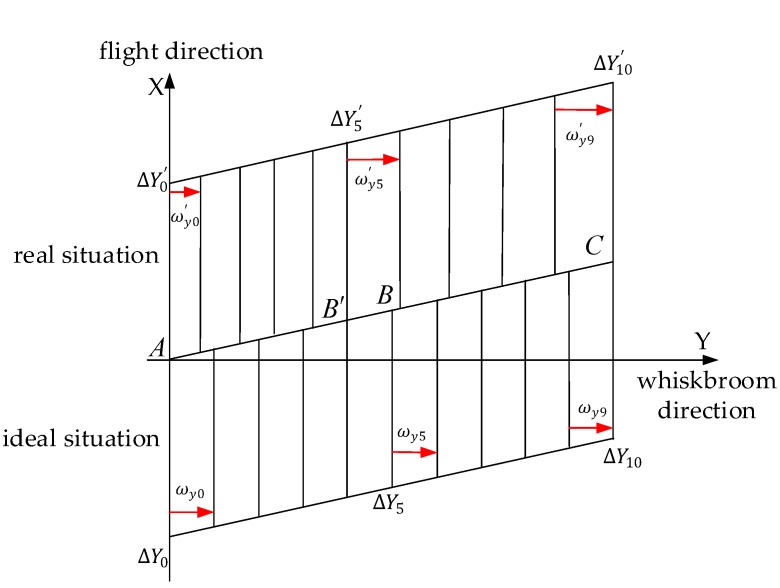
A ground coverage map with equal angles and unequal angles.

Figure 5 shows the image of ground coverage in the ideal case (equiangular whiskbroom) and in the actual case (unequal angle). Ideally, because of the uniform whiskbroom, the whiskbroom angle $\varphi_{0}\;\sim \;\varphi_{10}$ for each line is the same with a uniform coverage of the ground. In reality, when the whiskbroom velocity is less than the set value, the whiskbroom angle $\varphi_{0}^{\prime }\;\sim \;\varphi_{5}^{\prime }$ for each line is less than the set value of equiangular whiskbroom, and at that moment the image cannot be formed at *B.* However, at *B’*, when the whiskbroom velocity is greater than the set value, the whiskbroom angle $\varphi_{5}^{\prime }\;\sim \;\varphi_{10}^{\prime }$ for each line is greater than the set value of the equiangular whiskbroom, thus the image is formed at *C* and total cumulative error of displacement $\Delta Y_{0}^{\prime }\;$~$\;\Delta Y_{10}^{\prime }$ is 0. As can be seen from Figure 5, the offset value caused by an unequal angle whiskbroom is only distributed across the track.

#### 2.3.1. The Calibration of the Imaging Start Angle

The distortion caused by unequal angular whiskbroom is significant [19]. Therefore, the error of the image plane across the track can be directly used as the observed value to calibrate the imaging start angle. Supposing that in the vertical direction of the original thermal-infrared image, the difference between the pixel of the first whiskbroom line and the same pixel of the control image is Δy. When the whiskbroom angle velocity of the first whiskbroom line is set as $\omega_{y0}$, the ending whiskbroom angle of the first whiskbroom line will be:

(7) $$\varphi_{img1}\;=\;\Delta y\cdot \omega_{y0}\cdot \tau ,$$

Since the whiskbroom angle is related with the imaging start angle of each image in the same way as the calibration for the imaging time start, the angle calibration needs to be done from the first image. Since the whiskbroom angle of the first imaging line is less than 10^−5^°, it can be approximated as $\varphi_{img1}$ = $\varphi_{img0}$.

#### 2.3.2. The Calibration of the Line Angular Velocity

According to the principle of using detector look angles to calibrate interior orientation elements, a segmented mode [20] is adopted to calibrate the whiskbroom angle of each line. That is, supposing that during a very short period of time, the angular velocity of each scan line is constant, the total number of scan lines $L_{wsk}$ is divided into *m* segments on average. Thus, the whiskbroom lines of each segment is $L_{wsk}$/m and the ground displacement across the track ΔY is converted to the image as:

(8) $$\Delta Y\;=\;A_{y}\cdot \frac{\omega_{ym}}{\cos\gamma_{y}}\frac{L_{wsk}}{m}\cos K_{zy},$$

In Equation (8), $A_{y}$ is the mapping scale of the image across the track; $\omega_{ym}$, the velocity of the whiskbroom angle in segment *m*; $\gamma_{y}$, the component of the drift angle across the track; and *K_zy_*, the across-the-track component of the mounting angle of the camera detector around the *Z* axis. In two consecutive images, the displacement across the track is $\Delta Y\;=\;\left|y_{2}-y_{1}\right|$. Δ$\omega_{ym}$ and Δ$K_{zy}$, the correction value of $\omega_{ym}$ and $K_{zy}$, are set as an unknown number to establish error equations and the normal equation in order to get the least-square solutions, thus getting the whiskbroom angle velocity calibration value in each segment so as to get the whiskbroom angle of each whiskbroom line.

### 2.4. The Spaceborne Linear Array Imaging Geometric Model

According to the imaging geometry model of the pushbroom camera [21,22,23], with the characteristics of pushbroom camera taken into consideration, the imaging geometric model of the linear array whiskbroom camera’s geometric calibration is established as follows:

(9) $$\left[\begin{array}{c} X\\ Y\\ Z \end{array}\right]\;=\;\left[\begin{array}{c} X_{s}\left(t\right)\\ Y_{s}\left(t\right)\\ Z_{s}\left(t\right) \end{array}\right]+mR_{J2000}^{WGS84}R_{body}^{J2000}\left(t\right)R_{camera}^{body}R_{U}R_{S}\left(t\right)\left[\begin{array}{c} l_{x}\\ l_{y}\\ 1 \end{array}\right]\;R_{S}\left(t\right)\;=\;Rot\left(\varphi_{img}\left(t\right),0,0\right)\;=\;\left[\begin{array}{ccc} 1 & 0 & 0\\ 0 & \cos\varphi_{img}\left(t\right) & \sin\varphi_{img}\left(t\right)\\ 0 & -\sin\varphi_{img}\left(t\right) & \cos\varphi_{img}\left(t\right) \end{array}\right],$$

In Equation (9), [*X Y Z*]^T^ is the coordinate of an object space point in the WGS84 coordinate system; [*X_S_ Y_S_ Z_S_*]^T^, the position vector of the satellite in the WGS84 system while imaging in orbit; $R_{J2000}^{WGS84}$, the rotation matrix between the J2000 coordinate system and the WGS84 coordinate system; $R_{body}^{J2000}$, the rotation matrix between the body coordinate system and the J2000 coordinate system, which has been measured and processed by the setting equipment (star sensor); $R_{camera}^{body}$, the rotation matrix of camera installation angle in the body coordinate system; and $R_{U}$, the offset matrix. Because of the factors such as the satellite orbit determination errors, attitude measure errors, installation errors of image loading, and so on, the system error is caused by the satellite image positioning. The offset matrix is directly used to correct the directional deviation between the imaging ray and the actual ray with (*lx*, *ly*)^T^, in which the detector look angle *s* is represented by a three polynomial model, with the direction angle of each pixel in the camera coordinate system comprehensively representing internal orientation elements and their distortion, with *m* as the scale factor. $R_{S}\left(t\right)$ is the rotation matrix composed of the scanning mirror around the *X* axis of the sensor coordinate system. *R**_U_*** and (*lx*, *ly*)^T^ are used to conduct external calibration and internal calibration, respectively. Except for $R_{S}\left(t\right)$ and *t*, which are used for whiskbroom calibration, the geometric model of the others is the same as that of the spaceborne linear array pushbroom imaging.

## 3. Experiments and Results

The experiment uses the YG-14 thermal-infrared images of Anyang and Taiyuan in China, with a spectral band of 7.62 ~ 10.20 μm and a focal length of 245.7 mm. Digital Orthophoto Maps (DOM) of 1:5000 and Digital Elevation Models (DEM) in the corresponding regions are taken as control data. The Anyang data is applied to geometric calibration, and the Taiyuan data is used for the calibration verification experiment. External calibration takes five control points by manually produced points. In order to study the law of error distribution, the whiskbroom calibration and the internal calibration take tens of thousands of checkpoints by means of the image matching method, with a matching accuracy within 0.3 pixels, uniformly distributed. The experimental data sets are shown in Table 1.

### 3.1. External Calibration and Analysis

**Figure 6:**
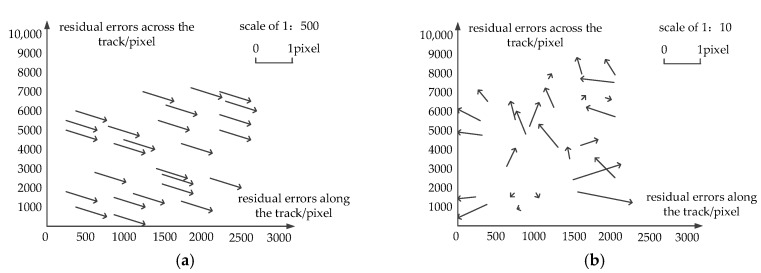
Residual distribution of the check points: (**a**) Residual distribution of direct positioning; (**b**) Residual distribution after external calibration.

The exterior orientation accuracy, by use of the offset matrix method, is shown in Table 2. As seen from it, the direct positioning accuracy is poor because—influenced by the force in the process of satellite launching—the camera mounting matrix changes in orbit. As shown in Figure 6, after external calibration, the residual errors of check points are no longer systematic, and meanwhile, the accuracy of geometric calibration improves remarkably, with the accuracy along the track being better than 4 pixels and that across the track being better than six pixels. Obviously, the offset matrix compensates for the systematic error of camera installation and attitude measurement, improving the positioning accuracy of the linear array whiskbroom camera without GCP.

**Figure 7:**
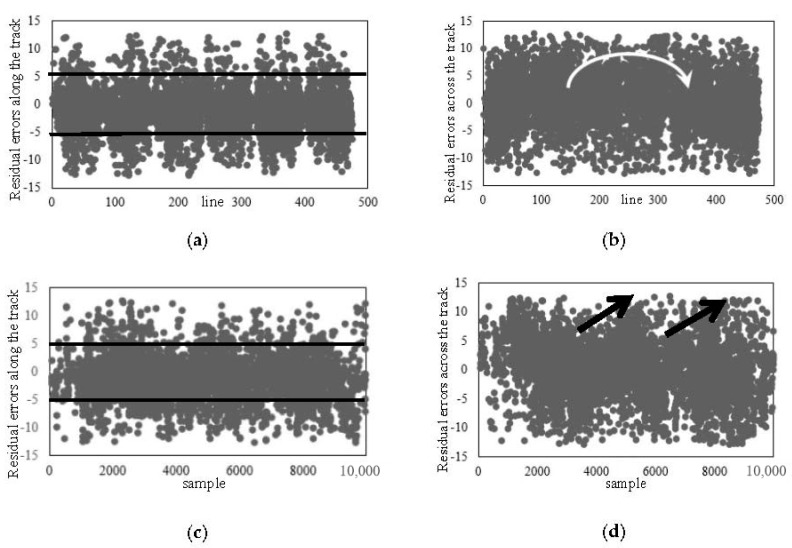
Residual errors after external calibration: (**a**) Residual errors along the track (flight direction); (**b**) residual errors across the track (flight direction); (**c**) residual errors along the track (whiskbroom direction); (**d**) residual errors across the track (whiskbroom direction).

Figure 7 illustrates the residual errors after external calibration. In Figure 7a,c, the horizontal coordinates are image lines and columns, and the vertical coordinates are the residual errors along the track, with the numerical value along the track being significantly negative because the whiskbroom imaging cycle is inaccurate. In Figure 7b,d, the horizontal coordinates are image lines and columns, and the vertical coordinates are the residual errors across the track. As shown in Figure 7d, the residuals across the track have obvious continuous fluctuations in the direction across the track. As indicated by the arrow in the continuous phase, the error is caused by the accumulation volume of the whiskbroom angle error, and this has verified the rule of the distortion caused by the uneven whiskbroom motion. As seen from Figure 7b, the residuals along the track takes on a trend of “two large ends, a small middle” and as indicated by the arrow, this is caused by the internal orientation element error of the camera. 

### 3.2. The Calibration Test and Analysis of Whiskbroom Time

**Figure 8:**
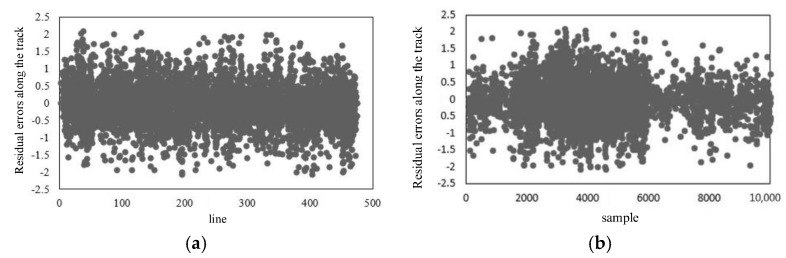
Residual errors after the calibration of the temporal system parameters: (**a**) Residual errors along the track (flight direction); (**b**) residual errors across the track (flight direction).

Figure 8 shows the distribution of residual errors after the calibration of the temporal time system parameters. As shown in Figure 7 and Table 2, the residuals along the track are reduced to be better than 2.5 pixels. Meanwhile, as seen from the contrast with Figure 7a,c, their distribution along the track is uniform globally. It has been verified that in the imaging geometric model that has been calibrated by the temporal time system parameters, the error along the track, caused by the inaccuracy of the imaging time and the line integration time, has been eliminated. 

### 3.3. The Calibration Test and Analysis of Whiskbroom Angle

**Figure 9:**
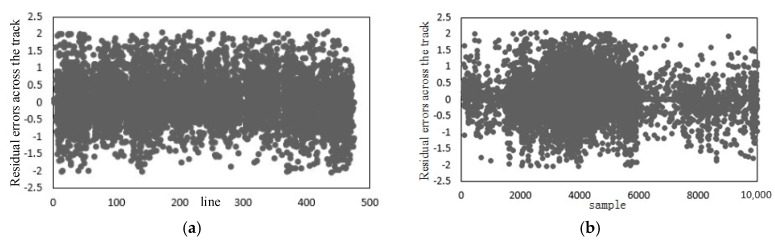
Residual errors after the calibration of the whiskbroom angle parameters: (**a**) Residual errors along the track (whiskbroom direction); (**b**) residual errors across the track (whiskbroom direction).

Figure 9 is the distribution of residual errors after the calibration of the whiskbroom angle parameters. As shown in Figure 9 and Table 2, the residuals after the calibration of the whiskbroom angle parameters across the track are reduced to less than 2.5 pixels. Meanwhile, as seen from the contrast with Figure 7b,d, on the one hand, the residuals across the track no longer show local regularity and their distribution is uniform globally, and on the other hand, as shown in Figure 9a, there is still, on the whole, obvious errors in the vertical coordinates along the track, taking on a trend of “two large ends, a small middle”.

### 3.4. Internal Calibration Test and Analysis

**Figure 10:**
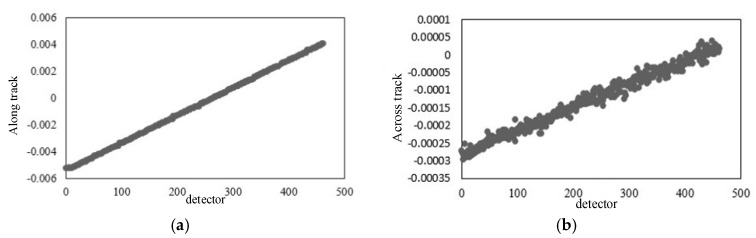
The diagram of detector look angle: (**a**) Along the track; (**b**) across the track.

In Figure 10a,b, the horizontal coordinate is the detector and the vertical coordinate is the detector look angle, along and across the track respectively, showing its true arrangement. This shows that the error across the track, caused by the non-uniform scanning angle, has been effectively eliminated, but there still exists an interior orientation element error. 

**Figure 11:**
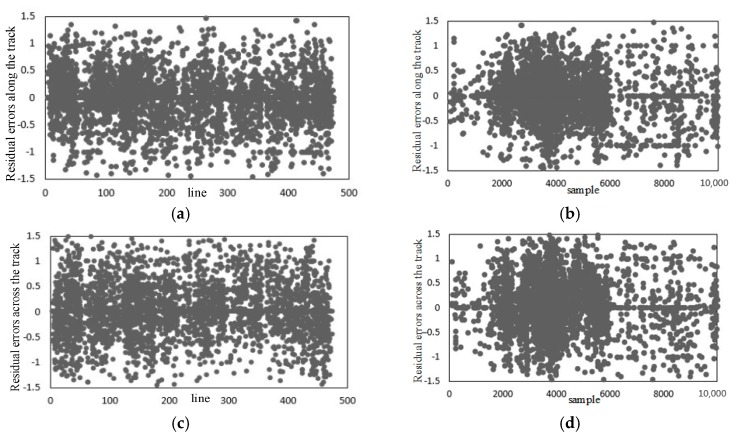
Residual errors after internal calibration: (**a**) Residual errors along the track (flight direction); (**b**) residual errors across the track (flight direction); (**c**) residual errors along the track (whiskbroom direction); (**d**) residual errors across the track (whiskbroom direction).

Figure 11 is the graph of the distribution of the residual errors after internal calibration. It can be seen from the contrast with Table 2 and Figure 8 and Figure 9, that after internal calibration the interior orientation element error in the camera has also been eliminated. Additionally, either along the track or across the track, there is no obvious regularity for the residual error in the imaging geometric model globally. Besides this, the distribution of the residuals is uniform and the residual error is better than 1 pixel.

### 3.5. Splicing Test and Analysis

**Figure 12:**
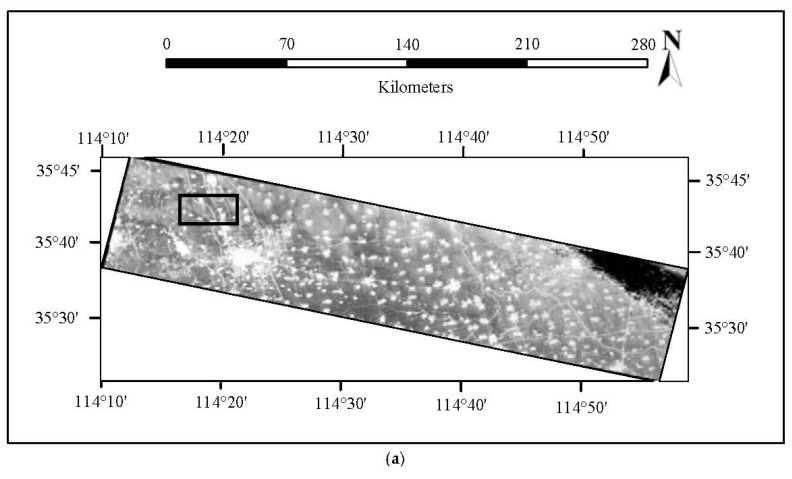

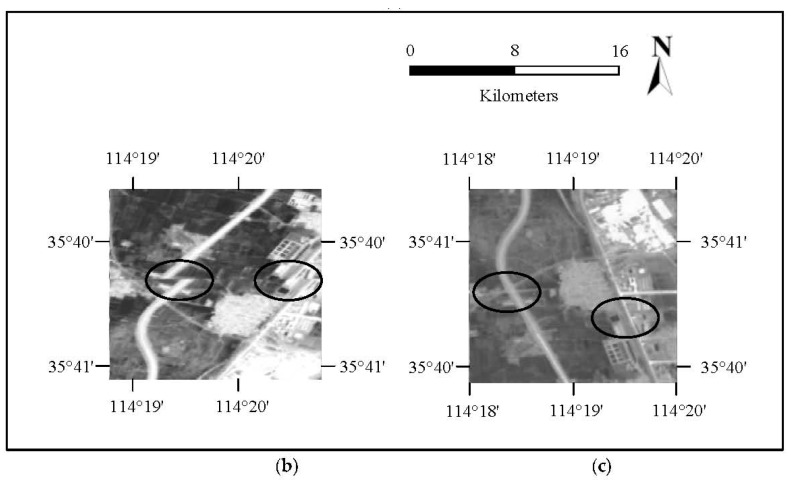
The mosaic result of four image sequences: (**a**) Panorama mosaic image after geometric calibration; (**b**) local mosaic result of original images; (**c**) local mosaic result after geometric calibration.

The four image sequences without GCPs are spliced after geometric calibration, as shown in Figure 12a, and their details are enlarged, as shown in Figure 12c. Figure 12b is the graph of the enlarged details of the image mosaic without calibration. It can be seen from the contrast between Figure 12b,c that there is an obvious dislocation at the joint part of the image mosaic without calibration on the road and in the area of buildings. However, there is almost no visual dislocation of the image mosaic after geometric calibration in the connection area. 

### 3.6. Geometric Calibration Compensation Test

**Figure 13:**
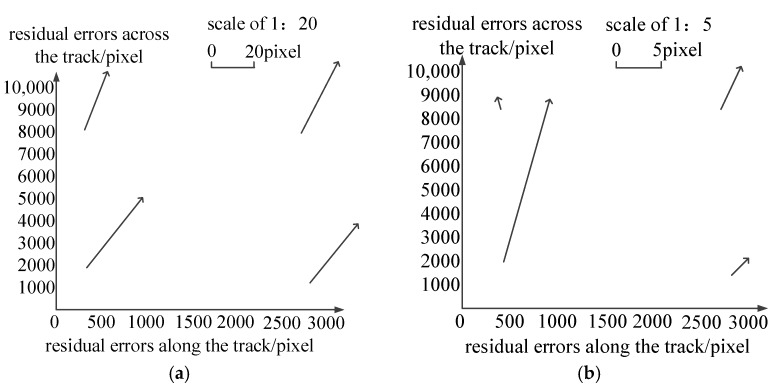
Residual distribution of the check points: (**a**) Residual distribution of calibration offset; (**b**) residual distribution parameter compensation (without GCP).

The calibration parameter obtained by the method proposed in this paper is used to compensate for the thermal-infrared images in Taiyuan, China, and the results are shown in Figure 13. The plane accuracy after compensation by the use of the offset matrix is better than 30 pixels, indicating that the offset matrix has compensated for the systematic errors of camera installation and attitude measurement, improving the positioning accuracy. After the compensation of the calibration parameter, the plane accuracy is better than 9 pixels, verifying the reliability of the geometric calibration results.

## 4. Discussion

Aimed at the geometric calibration of the spaceborne linear array whiskbroom camera, this paper has proposed a spaceborne linear array whiskbroom imaging geometric model that makes use of the offset matrix method and the modeling method of detector look angle to calibrate exterior orientation elements and interior orientation elements. For the time uncertainty caused by the whiskbroom, the time system calibration is conducted by converting the object surface displacement to the image plane in order to set the time start and the line integration time. Furthermore, for the angle uncertainty caused by the whiskbroom, the segmentation method is used to discretize the continuous whiskbroom angle to calibrate the whiskbroom angle so as to set the imaging start angle and the line whiskbroom angle velocity. 

The validity and reliability of the method proposed in this paper has been tested and verified. After the external calibration, the numerical value of the residual errors along the track is significantly negative because of the inaccuracy of the whiskbroom imaging cycle, and the residuals across the track have obvious continuous fluctuations due to the accumulation volume of the whiskbroom angle error. With the time system calibrated, the residual errors along the track are reduced to no more than 2.5 pixels, resulting in a uniform distribution of residuals in the whole area. This indicates that the errors along the track, which are caused by the inaccuracy of the imaging time start and the line integration time, have been eliminated. Additionally, with the whiskbroom angle calibrated, the residual across the track are reduced to no more than 2.5 pixels, and the residuals across the track which are typical of “two large ends, a small middle”, have been eliminated. This indicates that the errors across the track, which are caused by the inaccuracy of the imaging start angle and the whiskbroom angular velocity, have also been eliminated. With the internal calibration done, the accuracy of the imaging geometric model has been further improved, and there is no visual dislocation of the image mosaic in image sequences, therefore verifying the validity of the method of geometric calibration proposed in this paper. With the calibration parameters compensated, the plane accuracy is better than 9 pixels, verifying the reliability of geometric calibration results. 

Admittedly, the method of geometric calibration proposed in this paper effectively improves the accuracy of the geometric positioning of the linear array whiskbroom camera and also eliminates the image distortion, but there are some shortcomings to the method. In this paper, the error of exterior orientation elements, the error of the time system, the error of the whiskbroom angle, and the error of the interior orientation elements are calibrated one by one, and all these errors are believed to be independent from each other. In reality however, the sources of the errors caused by the linear whiskbroom motion are complicated, and the effects of errors may overlap with each other, and as a result, affect the stability of the imaging geometric model. Therefore, it is necessary to further analyze the control mechanism of the whiskbroom of the scanning mirror and strictly categorize the sources of errors so as to get an imaging geometric model that is of more stability.

## 5. Conclusions

This paper has analyzed the errors caused by the motion of the whiskbroom, pointing out that the imaging time start, the line integration time, the imaging start angle, and the line whiskbroom angular velocity are the keys to the geometric calibration of the whiskbroom camera. Directed against the track deviation caused by using equiangular velocity in the airborne linear array whiskbroom system, we considered the non-equiangular whiskbroom characteristics in the spaceborne whiskbroom system, calibrating the imaging start angle and line whiskbroom angular velocity. By calibrating the non-uniform whiskbroom angle, the error across the track is overcome. The results of the experiment are shown as follows. Firstly, the external calibration method of the pushbroom camera is suitable for the whiskbroom camera and the offset matrix is able to compensate for the systematic errors from the on-orbit imaging load mounting, the attitude and orbit measurements, and so on, so as to improve the positioning accuracy of the imaging without GCPs. After the compensation of the offset matrix, the plane accuracy of the YG-14 thermal-infrared image without GCPs is better than 9 pixels. Secondly, with the whiskbroom calibrated, the positioning accuracy along the track with GCPs is better than 2.5 pixels in addition to the uniform distribution of the residual errors and the improvement of the internal stability of the model. The positioning accuracy across the track with GCPs is better than 2.5 pixels, with the numerical value of the residual errors being lower than the external calibration results, and besides this, the residual errors no longer show global regularity. Thirdly, with the interior orientation elements calibrated, the plane positioning accuracy of the YG-14 thermal-infrared image with GCPs is better than 1 pixel, and the detector look angle can comprehensively indicate the interior orientation elements and their distortions.

## Figures and Tables

**Table 1:** Experimental data sets.

Region	Time		Topography
	Control Images	Thermal-Infrared Images	
Anyang	2012	2014–06	plain, slight elevation variation
Taiyuan	2008	2014–04	including hills, elevation variation 700 m

**Table 2:** Geometric calibration accuracy of the thermal-infrared linear array whiskbroom camera in the YG-14 (Pixel).

Positioning Accuracy	Min	Max	RMS	Min	Max	RMS
Direct Positioning	40.9873	50.2916	45.7530	4.2630	37.7448	25.9728
External Calibration	0.0008	14.7900	3.2898	0.0027	14.8636	5.5814
Whiskbroom	0.0003	12.0003	2.2948	0.0011	12.0012	4.7623
Temporal System Calibration						
Whiskbroom	0.0001	10.6120	1.9273	0.0004	9.4169	2.1894
Angle Calibration						
Internal Calibration	0.00023	1.4526	0.4209	0.000009	1.4787	0.4671
